# Morphological landscape of endothelial cell networks reveals a functional role of glutamate receptors in angiogenesis

**DOI:** 10.1038/s41598-020-70440-0

**Published:** 2020-08-14

**Authors:** Heba Z. Sailem, Ayman Al Haj Zen

**Affiliations:** 1 Institute of Biomedical Engineering, Department of Engineering Science, University of Oxford, Old Road Campus Research Building, Oxford, OX3 7DQ UK; 2 Big Data Institute, Li Ka Shing Centre for Health Information and Discovery, University of Oxford, Old Road Campus Research Building, Oxford, OX3 7LF UK; 3 College of Health and Life Sciences, Hamad Bin Khalifa University, Education City, Doha, Qatar; 4 Radcliffe Department of Medicine, British Heart Foundation Centre of Research Excellence, University of Oxford, Oxford, UK

**Keywords:** Image processing, Cellular imaging, Drug discovery

## Abstract

Angiogenesis plays a key role in several diseases including cancer, ischemic vascular disease, and Alzheimer’s disease. Chemical genetic screening of endothelial tube formation provides a robust approach for identifying signalling components that impact microvascular network morphology as well as endothelial cell biology. However, the analysis of the resulting imaging datasets has been limited to a few phenotypic features such as the total tube length or the number of branching points. Here we developed a high content analysis framework for detailed quantification of various aspects of network morphology including network complexity, symmetry and topology. By applying our approach to a high content screen of 1,280 characterised drugs, we found that drugs that result in a similar phenotype share the same mechanism of action or common downstream signalling pathways. Our multiparametric analysis revealed that a group of glutamate receptor antagonists enhances branching and network connectivity. Using an integrative meta-analysis approach, we validated the link between these receptors and angiogenesis. We further found that the expression of these genes is associated with the prognosis of Alzheimer’s patients. In conclusion, our work shows that detailed image analysis of complex endothelial phenotypes can reveal new insights into biological mechanisms modulating the morphogenesis of endothelial networks and identify potential therapeutics for angiogenesis-related diseases.

## Introduction

The primary function of microvascular networks across the body is to provide nutrients and oxygen supply for tissues. Both the vasculogenic and angiogenic processes are coordinated to form the network of tissue microvasculature. Vasculogenesis is the de novo formation of vessels from the assembly of progenitors or mature endothelial aggregates^1^. While in angiogenesis, the new vessels are formed from pre-existing ones^2^. Endothelial cell branching and tubulogenesis are critical elements for the formation of functional microvascular networks during embryonic development and postnatal life. Defects in vascular network structure or formation play a significant role in many pathological conditions, including ischemic vascular disease, cancer, neurodegenerative diseases, inflammatory disorders, and diabetes^3^. Consequently, the identification of small molecules or signalling components that regulate this complex process has important implications for many diseases.

The features of the nascent endothelial network are determined by genetic components and the surrounding microenvironment. Changes in these factors can affect vessel morphology, structure, organisation, or permeability leading to different pathologies^3,4^. For instance, the vessels that are formed in tumours are often abnormal and can have various phenotypes, including irregular organisation, loosely assembled vessel wall, abnormally wide or thin vessels, and tortuous serpentine-like morphology^4^. On the other hand, leaky vessels have been observed in diabetic retinopathy and neurodegenerative diseases^3,5^. Furthermore, increased angiogenesis has also been implicated in Alzheimer’s disease where it has been shown that the formed vessels are structurally and functionally defective^6^. These changes include thinner and coiled vessels and increased density in mouse models^7^. Consequently, this can lead to ﻿inflammation and tissue damage due to impaired blood flow, compromised blood brain barrier and reduced nutrient delivery^6,7^. Therefore, developing methods for quantifying various aspects of endothelial networks is important for decoding the signalling components contributing to different vascular morphology^4^.

Endothelial cells have a remarkable ability to autonomously form tube networks, even in cell cultures. The presence of vascular growth factors and appropriate extracellular matrix substrate induce a specific response in endothelial cells to assemble and form branched tubes. For instance, within a few hours of being cultured on Matrigel, a natural basement membrane matrix, endothelial cells form a capillary-like network^8^. This network shares many aspects with in vivo microvascular networks such as the vascular branching, tubulogenesis, mesh formation, and structure complexity. However, aspects such as tree-like hierarchical branching structure and lumen formation are not represented. At the cellular level, endothelial networks recapitulate many biological processes of in vivo angiogenesis including cell–cell interaction, elongation, motility, and cell–matrix adhesion. Therefore, the changes detected in endothelial network morphology reflect molecular and cellular functions that propagate to specify tissue-level phenotypes.

Phenotype-based screening has been successful in identifying small molecules targeting a specific biological marker or process such as migration, proliferation, viability, or branching^9–12^. It has been shown that monitoring morphological changes at the cellular level can confer predictive insights on the drug mechanisms of action and its effect on cell biology^13^. Detailed morphology analysis further allows the detection of potential side effects, which ultimately can reduce the attrition rate of drug development pipelines and result in identifying more relevant therapeutic targets^14^. Furthermore, profiling the heterogeneity of cellular responses upon exposure to drugs has been shown to be useful for further characterisation of the drug mechanism of action^15,16^. The screening of more complex cell culture models that mimic in vivo biology is starting to emerge as these models are expected to select drugs that are more likely to mature in the clinic^17,18^. Although vascular morphogenesis assays have been established^19,20^, a comprehensive analysis of these complex culture models is still lacking. Moreover, the extent that higher level tissue properties reflect those at the molecular level is yet to be established.

**Figure 1:**
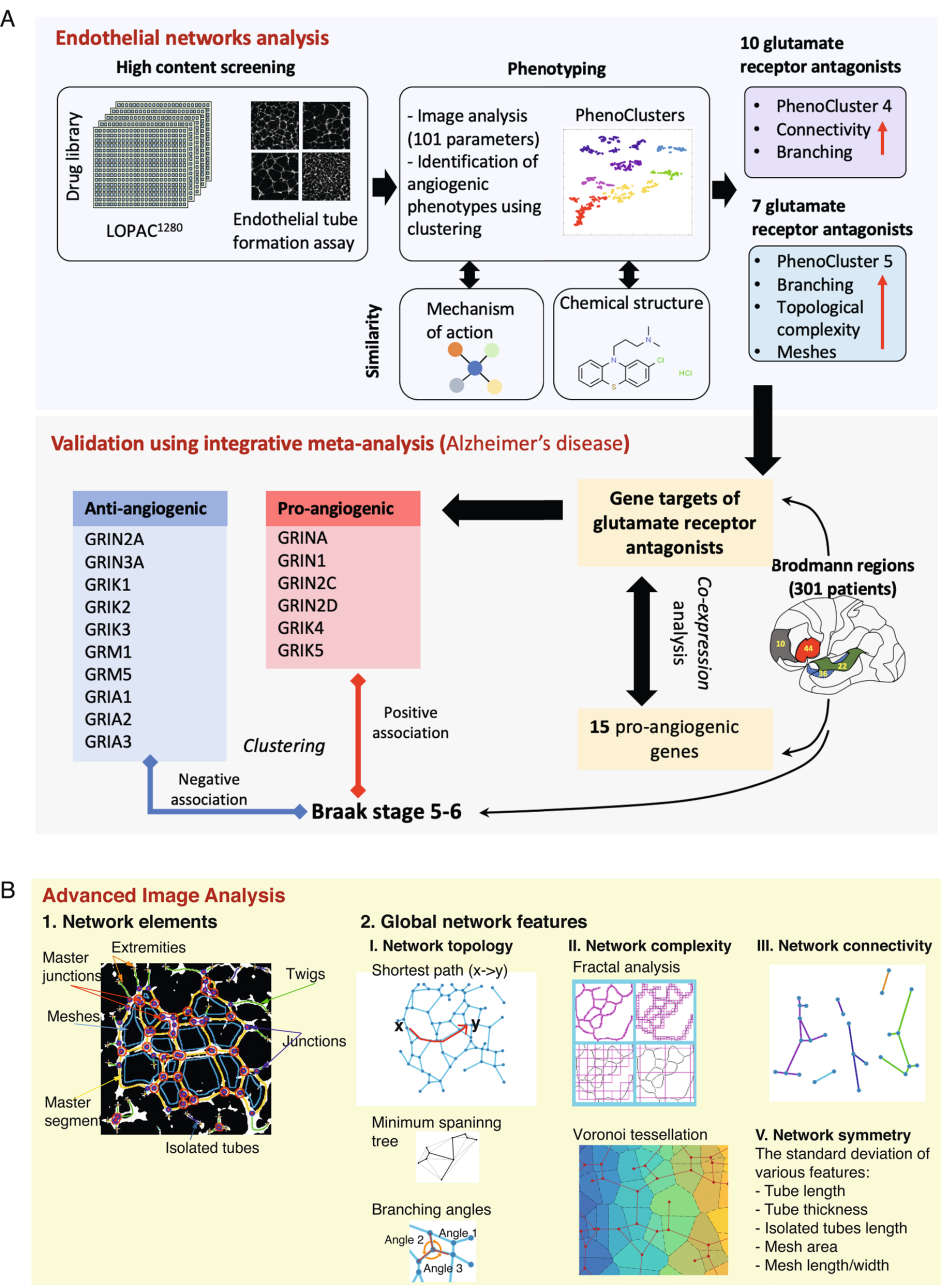
The outline of our integrative image-omic approach and the developed image analysis pipeline. (**A**) Overview schematic of the study. (**B**) Image analysis pipeline involves network detection, classification of network elements and feature extraction. Representation of the measured features which includes network topology, symmetry and complexity.

We propose that detailed phenotyping of endothelial network features in chemical genetic screening can aid the identification of drug mechanisms of action and elucidate genetic components driving this complex process. Specifically, we developed a computer vision method for quantifying complex phenotypic aspects of endothelial networks such as branching, connectivity, topology, and symmetry. To our knowledge, many of the proposed measures are novel and have not been investigated before. This enabled in-depth angiogenic phenotype characterisation of drugs in the LOPAC_1280_ library that have diverse mechanisms of action and target most signalling pathways^9^. Importantly, multivariate analysis revealed a novel anti-angiogenic role for a group of glutamate receptors based on the network phenotype of their chemical inhibition by glutamate receptor antagonists. Because glutamate receptors are involved in the neuropathogenesis of several diseases such as Alzheimer’s disease, these results were validated using an orthogonal clinical dataset measuring gene expression in Alzheimer’s Disease patients. This showed that the expression of these genes negatively correlates with angiogenic genes and their suppression correlates with worse patient prognosis. Therefore, these genes might provide a promising target in Alzheimer’s disease. Figure 1A shows a summary of the study. Altogether, our results show that multi-parametric profiling of vascular networks can not only advance our understanding of signalling components involved in the multistep endothelial network formation process but also holds the promise of discovering novel therapeutic targets.

## Results

### Identifying multi-dimensional phenotypic signatures of endothelial network formation

To comprehensively profile endothelial network patterns upon drug treatments, we used a dataset from a previous high content screen of the LOPAC_1280_ small molecule library^19^. In the previous study, 15 enhancers and 79 inhibitors of tube formation were identified where the analysis has been limited to one feature of the network (total tube length). Despite the importance of this parameter as a descriptor of network formation, single parameter analysis does not account for other aspects of endothelial network morphology. We classified network elements as tubes, nodes (i.e., branching points), or meshes (area enclosed within tubes) (Fig. 1B). Tubes are further sub-classified into twigs, segments, and isolated tubes based on their connectivity while nodes are subclassified into junctions, master junctions, and extremities (Methods). In total, we extracted 101 features quantifying tube formation (e.g., number of tubes and total tube length), tube morphology (e.g., mean tube length and thickness), mesh formation (e.g., number of meshes, mean mesh area, and total mesh area), and network connectivity based on the number of connected components and isolated tubes. Furthermore, we propose and develop various global measures of the formed network including: network symmetry (homogeneity of network elements across the well which can be quantified using the Standard Deviation (SD) of various elements), network complexity based on fractal analysis and Voronoi tessellation, and network topology based on network centrality measures from graph theory such as the average length of the shortest paths between nodes and minimum spanning tree (Fig. 1B, Supplementary Table 1 and Methods).

**Table 1:** Enrichment of high Braak stage patients (stage 5 and 6) in various patient clusters and brain regions based on Fisher Exact Test.

Region	Cluster P1—*p*-value	Cluster P2—*p*-value	Cluster P3—*p*-value
BM10	0.2207	0.9988	0.0075
BM22	0.8736	0.9969	0.0007
BM36	0.1611	1.0000	0.0007
BM44	0.9709	0.1221	0.1137

**Figure 2:**
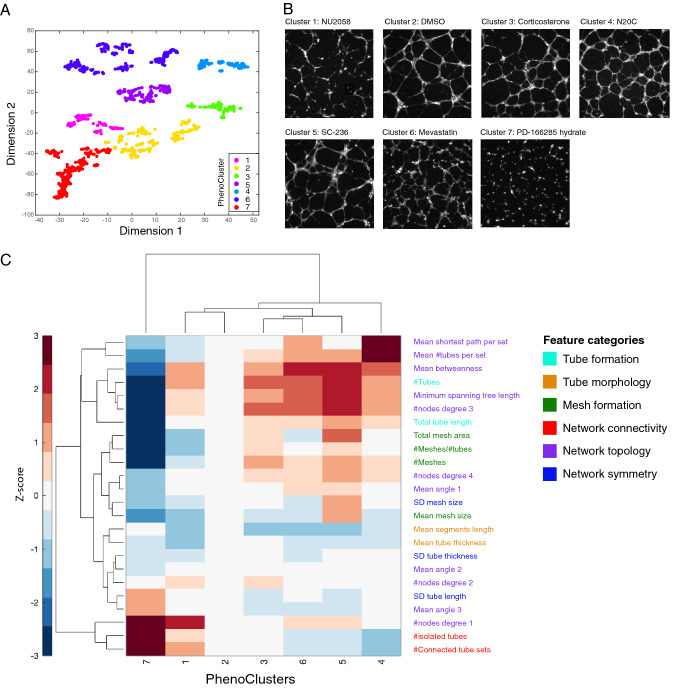
Quantitative analysis of endothelial networks reveals distinct morphological phenotypes. (**A**) Clustering and visualisation of LOPAC compounds in t-SNE space based on 101 phenotypic features. (**B**) Representative images for each PhenoCluster. (**C**) Quantitative differences between various clusters based on a representative set of features from different categories.

Many drugs affected multiple aspects of endothelial network formation. Hierarchical clustering was performed to group small molecules with similar phenotypic profiles, using Euclidean distance and ward linkage (Fig. 2A). This analysis revealed seven distinct phenotypic clusters (PhenoClusters), where PhenoCluster 2 is composed of control-like drugs that have no significant effect on network features. Other clusters exhibited distinct phenotypes ranging from changes in branching and mesh formation to changes in network topology (Fig. 2A,B and Supplementary Table 2). Drugs in PhenoCluster 1 and 7 are characterised by significant disruption of network formation. However, only drugs in PhenoCluster 7 significantly decreased tube formation by endothelial cells (Fig. 2B,C). Many of the drugs in this cluster are potent inhibitors of angiogenesis such as the positive control Suramin and VEGF inhibitors (DMH4, SU4312, SU5416 and Sunitinib) (Fig. 2C). Moreover, 95% of the drugs that were identified as inhibitors in the previous study^19^ are in this cluster (75/79 Fisher exact test; *p* < 1.45e-48).

Drugs in PhenoCluster 3, 4, 5, and 6 resulted in an enhancement of many tube and mesh formation features but varied in other features (Fig. 2C, Supplementary Fig. 1A,B). This increase in branching is also associated with improved connectivity in these clusters except for PhenoCluster 3 that instead exhibited an increase in the number of 2-way junctions (Supplementary Figure 1C–E, N). PhenoCluster 4 is characterised by the highest increase in connectivity as most tubes belong to the same network which is reflected by the significant decrease in the number of connected sets and the number of isolated tubes (Fig. 2C and Supplementary Fig. 1C–E). On the other hand, drugs in PhenoCluster 5 affected mesh formation where the total, average and standard deviation of mesh size are increased (Supplementary Fig. 1G–I). They also affected the network topology as the orientation of tubes in 3-way nodes (i.e., branching points with three tubes) is changed where the largest angle is increased, and the smallest angle is decreased (Supplementary Fig. 1J,K). The network in PhenoCluster 6 showed a decrease in average mesh area (Supplementary Fig. 1H) and increase in thin and short tubes which indicate immaturity of these tubes (Fig. 2B and Supplementary Fig. 1L,M). Taken together, these results illustrate that our multi-dimensional profiling allows distinguishing different morphologies of endothelial cell networks.

### Mapping endothelial network signatures to drug mechanism of action and chemical structure

**Figure 3:**
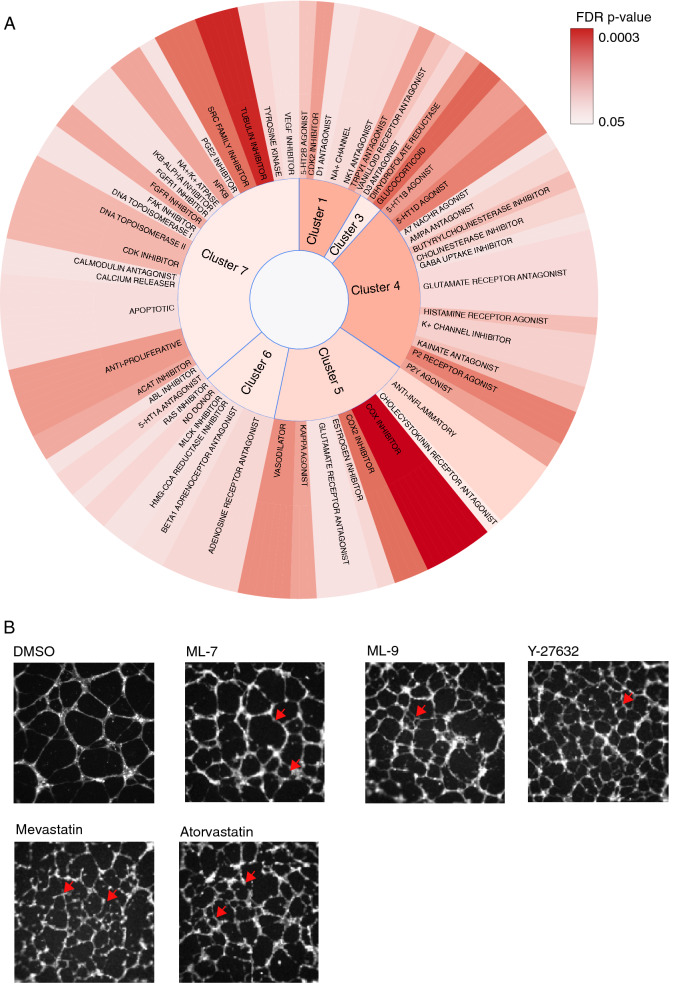
Endothelial PhenoClusters are enriched for various mechanisms of action. (**A**) For each cluster, the enriched mechanisms of actions are indicated proportional to their size. The significance of the enrichment is based on Fisher Exact test and corrected using false discovery rate. (**B**) Representative images of endothelial networks following treatment with MLCK inhibitors (ML-7 and ML-9), the ROCK inhibitor Y27632, or statins (HMGA reductase inhibitors: mevastatin and atrovastatin). Red arrows indicate characteristic features such as branches with altered morphology (thin and jaggy) and smaller mesh size.

To validate that our vascular signatures are biologically relevant, we performed enrichment analysis for biological mechanisms of action. We found that drugs in each PhenoCluster are significantly enriched for many mechanisms of action and signalling components (FDR *p*-value < 0.05) (Fig. 3A and Supplementary Table 3). For example, PhenoCluster 4 is enriched for many neurotransmission-related mechanisms of action. PhenoCluster 7, which is characterised by the complete disruption of tube formation, is composed of many drugs that interfere with processes that are required for endothelial cell proliferation and migration. At the molecular level, these include drugs that disrupt cytoskeletal reorganisation and cellular adhesion, such as tubulin and focal adhesion kinase inhibitors, and anti-proliferative drugs such as DNA topoisomerase I and II inhibitors. PhenoCluster 5 is enriched for anti-inflammatory mechanisms of action such as cyclooxygenase (COX) inhibitors and kappa agonists. These results confirm that our phenotypic features allow biologically relevant classification of drug effects on tube formation.

Many compounds in PhenoCluster 6, which is characterised by immature thin tubes, are associated with Myosin Light Chain (MLC) mechanism of action. These include the MLC Kinase (MLCK) inhibitors MLC-7 and MLC-9, as well as the ROCK inhibitor Y-27632 (Fig. 3B). The observed phenotype is consistent with MLCK role in cell motility, polarization, and adhesion^21^. Other myosin-related mechanisms that are enriched in this cluster are NO donor compounds (3 out of 4), Ras inhibitors (2 out of 2), and HMG-CoA reductase inhibitors (2 out of 2) (Fig. 3A). These results are in agreement with previous studies as NO production has been shown to inactivate MLCK^22^ and RAS has been shown to be required for MLCK activation^23^. Interestingly, the inhibition of HMG-CoA reductase has been shown to reduce endothelial cell migration due to its effect on RhoA localisation, which is upstream of MLCK^24^. The fact that drugs that target similar signalling pathways have a similar network signature, illustrate that tissue level features can provide a robust proxy for changes at the molecular level.

**Figure 4:**
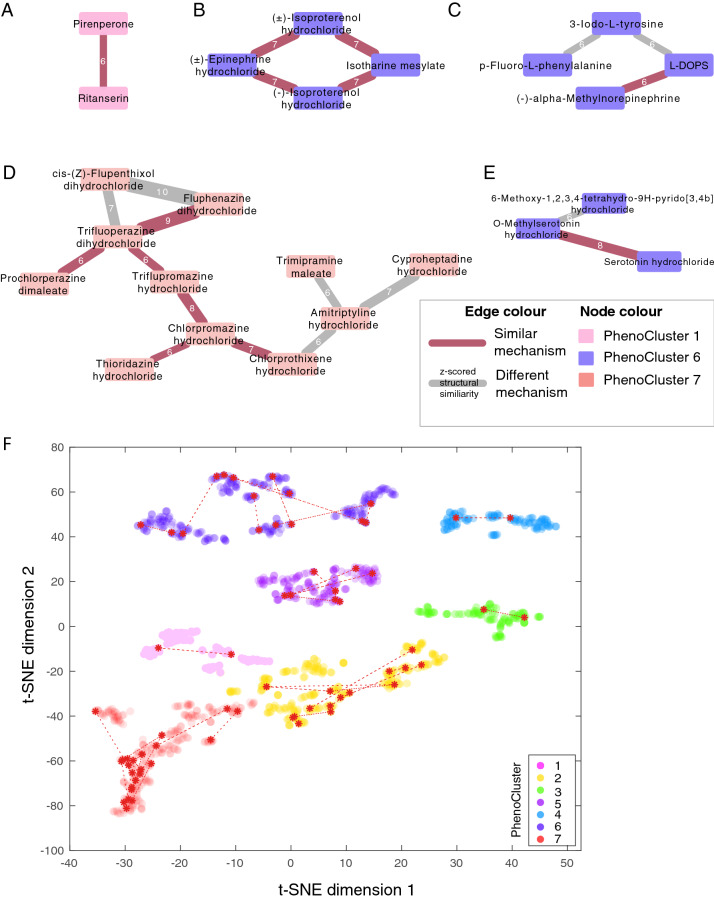
Structural similarity between compounds complements phenotypic analysis. (**A–E**) Structural similarity between compounds in the same cluster where drugs are represented as nodes and structural similarity based on Tanimoto distance as edges. Compounds are connected by a red edge if they share the same mechanism of action and a grey edge otherwise. The numbers on the edges indicate the z-scored structural similarities rounded to the nearest integer. Node colour indicates the compound cluster. (**F**) Compounds with a similar structure in the same PhenoCluster are connected by a dashed line.

Next, we determined chemical structural similarity between drugs in the same phenotypic cluster. Using Tanimoto distance^14^, we observe that some structurally similar drugs also have similar bioactivities and cluster together (Fig. 4, and Supplementary Table 4). For example, the 5-HT2 serotonin receptor antagonist ritanserin and pirenperone in PhenoCluster 1 are structurally similar (Fig. 4A). Additionally, four adrenoceptor agonists in PhenoCluster 6 shared similar chemical structures, including isoproterenol hydrochloride, isoetharine mesylate, and epinephrine hydrochloride (Fig. 4B). On the other hand, L-DOPS and (-)-alpha-methylnorepinephrine are other adrenoreceptor agonists in PhenoCluster 6 that are structurally distinct from the aforementioned adrenoreceptors but rather are structurally similar to the tyrosine hydroxylase inhibitor 3-Iodo-L-tyrosine (Fig. 4C). Therefore, our phenotypic clusters could, in some cases, be explained by structural similarity, bioactivity, or both, which highlight the importance of phenotypic profiling to identify drugs with similar effects on cell physiology.

Analysing structural similarity along phenotypic similarity allows identification of potential off-target effects or the mechanism of action of tested compounds. In particular, drugs with similar chemical structure and similar phenotypic profile but different mechanisms of action can reflect off-target effects^25^. For example, we found a group of structurally similar drugs in PhenoCluster 7, many of which are antagonists of dopamine, including triflupromazine hydrochloride, chlorpromazine hydrochloride, prochlorperazine dimaleate, and fluphenazine dihydrochloride (Fig. 4D). However, the serotonin receptor antagonist cyproheptadine hydrochloride, serotonin inhibitor trimipramine maleate, and adrenoceptor antagonist amitriptyline hydrochloride are structurally similar to the dopamine antagonists in this cluster. This might suggest an off-target effect for these drugs, which is consistent with the fact that these drugs can have nonspecific activity against dopamine^26^.

On the other hand, 6-methoxy-1,2,3,4-tetrahydro-9H-pyrido[3,4b] indole is a monoamine oxidase inhibitor (MOAI) that is structurally and phenotypically similar to two serotonin receptor agonists (Fig. 4E). The phenotypic similarity between these drugs is consistent with the fact that MOAI inhibits serotonin metabolism resulting in its accumulation and activation of serotonin receptors in what is known as serotonin syndrome^27,28^. Therefore, combining phenotypic and structural similarity can facilitate the determination of the likely affected mechanism of action of a drug in a specific biological context.

### Glutamate receptor antagonists result in distinct endothelial network morphology

A group of drugs in PhenoCluster 4 is significantly enriched for several mechanisms of action associated with glutamate neuroreceptors (FDR * p*-value < 0.05, Fig. 3A). This cluster showed mainly increased branching and connectivity (Fig. 2C and Supplementary Fig. 1A–E). In particular, 10 out of 42 glutamate receptor antagonists are in this cluster, three of which target both AMPA receptors and kainate receptors (FDR *p-*value < 0.05). A smaller group of glutamate receptor antagonists phenocopied cluster 5 (7 out of 42 compounds). Although drugs in this cluster resulted in increased branching, they also altered network topology and mesh formation (Fig. 2C). Notably, the glutamate receptor antagonist memantine, which is used for treating Alzheimer’s patients, is in PhenoCluster 5. These results suggest that changes in glutamate receptor activity could affect the formation of microvasculature networks.

Many mechanisms of action related to glutamate neurotransmission were also enriched in PhenoCluster 4 including GABA uptake inhibitors and P2Y receptor agonists (Fig. 3A). GABA is a product of glutamate in the brain^29^ while ATP-activated purinergic P2Y receptors have been shown to reduce the expression of AMPA receptors^30^. Furthermore, the serotonin agonists of both 5-HTR1B and 5-HTR1D receptors, but not 5HTR21B (PhenoCluster 1), are also enriched in this cluster. This is in line with the fact that activation of 5-HTR1 receptors inhibits glutamate and GABA release, while activation of 5-HTR2 receptors has the opposite effect^31^. Altogether, these results further support a role for mechanisms downstream of glutamate receptors in angiogenesis.

### Integrative meta-analysis reveals a link between glutamate receptors and angiogenesis in patients with Alzheimer’s disease

Since the role of Glutamate receptors in angiogenesis is not very well characterized, we sought to determine their importance in a relevant disease model. The fact that both glutamate receptors and angiogenesis are implicated in Alzheimer’s disease motivated us to investigate whether the association between glutamate receptor genes and Alzheimer pathogenesis can be partially due to their role in angiogenesis. We utilised a dataset measuring gene expression in 301 Alzheimer’s patients^7,32^. The dataset is composed of brain samples with different Braak stages (1–6), where stage 6 is the most severe form of the disease^33^. The dataset spans four Brodmann (BM) brain regions: frontal pole (BM10), superior temporal gyrus (BM22), parahippocampal gyrus (BM36), and inferior frontal gyrus (BM44). To define an angiogenic signature, we investigated a set of known pro-angiogenic genes that were previously described to be involved in cancer and Alzheimer’s pathogenesis^7,34^.

**Figure 5:**
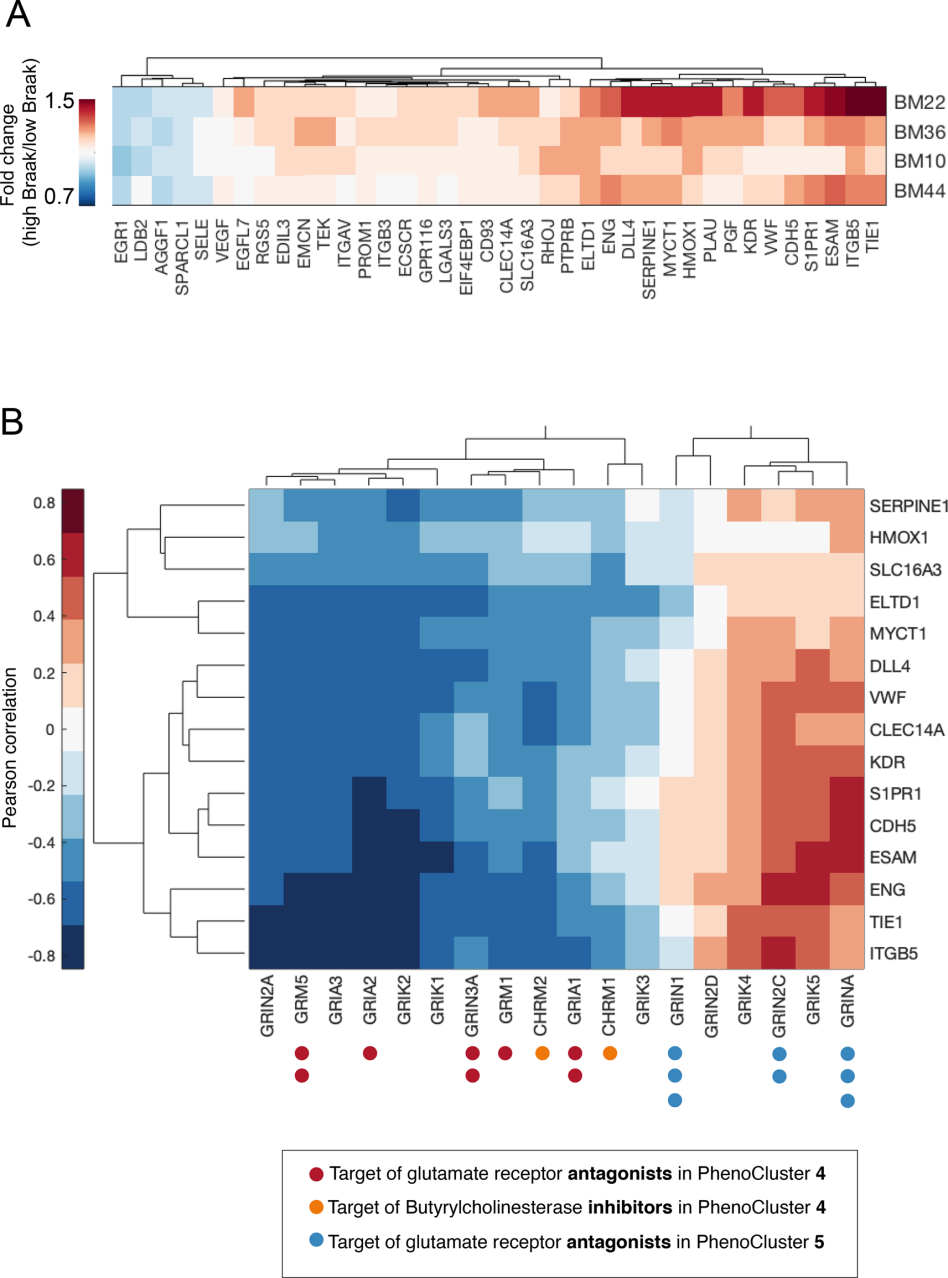
Association between glutamate receptors expression and angiogenesis in patients with Alzheimer’s disease. (**A**) Fold change of pro-angiogenic gene expression in Alzheimer’s patient with high Braak stage. (**B**) Pearson correlation between the expression of various glutamate receptors and pro-angiogenic genes that have higher expression in Braak stage 5 or more. The number of dots next to the genes represent the number of drugs that target these genes in the LOPAC library. Dot colour indicates the mechanism of action and PhenoCluster. Target genes of butyrylcholinesterase inhibitors that exhibited PhenoCluster 4-like phenotype were included for comparison.

First, we determined whether the selected set of pro-angiogenic genes are associated with patient outcome. We computed the fold change of expression values of these genes in patients with high Braak stage (5 and 6) versus low Braak stage (1 and 2) in different brain regions (Fig. 5A and Supplementary Fig. 2A). The classically affected superior temporal gyrus (BM22) and parahippocampal gyrus (BM36) regions show the highest fold-change in the expression of many pro-angiogenic genes. This is in accordance with a previous study ^7^. TIE1, ITGB5, ESAM, S1PR1, CDH5, VWF, KDR are among the genes with the highest expression fold-change in patients with high Braak scores (Fold change > 1.2, Kolmogorov–Smirnov * p*-value < 0.001). These results confirm a link between the expression of pro-angiogenic genes and the prognosis of Alzheimer’s patients.

Next, we determined the association between angiogenesis and glutamate receptors in Alzheimer’s patients. In this analysis, we included target genes of glutamate receptor antagonists in PhenoCluster 4, such as GRM5 and GRIN3A, as their chemical inhibition shows a “pro-angiogenic” phenotype (Methods and Supplementary Table 5). We also included target genes of butyrylcholinesterase inhibitors that induced PhenoCluster 4-like phenotype for comparison (Fig. 5B and Supplementary Table 5). We focused our analysis on the superior temporal gyrus region (BM22) as it showed the greatest change in the expression of pro-angiogenic genes. We observed that the expression of many glutamate receptors in PhenoCluster 4 is negatively correlated with the expression of pro-angiogenic genes (Pearson coefficient < − 0.3, and * p* value < 1.5e-05). Likewise, the expression of CHRM1 and CHRM2 genes, which are inhibited by the butyrylcholinesterase inhibitor ethopropazine hydrochloride, is also negatively correlated with the expression of pro-angiogenic genes (*p* value < 0.05). These results show that chemical genetic perturbations of genes that result in a similar network phenotype also have similar transcriptional profiles in patients, which further confirm the validity of our high content analysis. Moreover, these results further support an anti-angiogenic role for a group of glutamate receptor genes including GRM5 and GRIN3A.

On the contrary, the glutamate receptor genes GRIN1 and GRINA that are antagonized by drugs in PhenoCluster 5 were positively correlated with the expression of pro-angiogenic genes (Fig. 5B and Supplementary Table 5). Similar correlation patterns are observed in other brain regions except for the inferior frontal gyrus region (BM44) (Supplementary Fig. 2B–D). These results support a differential role of glutamate receptors in angiogenesis, which can have an important implication for Alzheimer’s disease.

**Figure 6:**
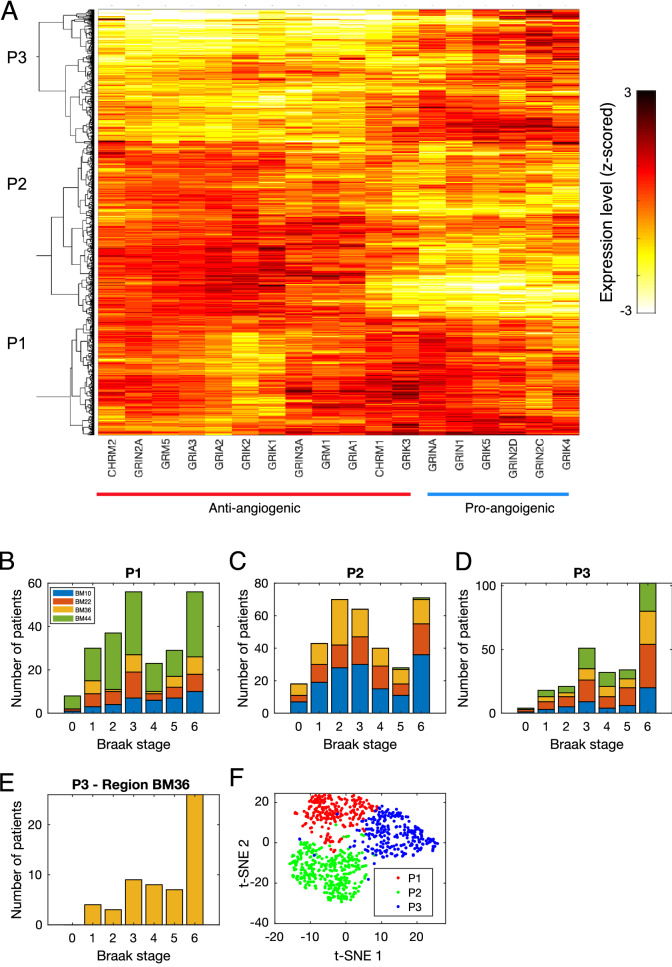
Glutamate receptors expression is associated with Alzheimer’s patient outcome. (**A**) Clustering of transcriptional profiles of glutamate receptors in different brain regions in Alzheimer’s patients shows three patient clusters. (**B**–**D**) Number of patients with different Braak stage in the different clusters. (**E**) Same as D but for brain region BM36. (**F**) Visualisation of transcriptional profiles in (A) using t-SNE.

In order to evaluate the link between the expression of glutamate receptors and patient outcome, we performed hierarchical clustering of Alzheimer’s patients based on the transcriptional profiles of glutamate receptor genes. We identified three main patient clusters: P1-P3 (Fig. 6A). Cluster P1 is enriched for transcription profiles of samples from the inferior frontal gyrus region (65.38% of BM44 profiles) (Fig. 6A,B). Most glutamate receptors have moderate to high expression in Cluster P1. On the other hand, the expression of anti-angiogenic glutamate receptors in Cluster P2 is high (Fig. 6A). This cluster is almost void of samples from BM44 region (Fig. 6C). In contrast, Cluster P3 exhibits a low expression of anti-angiogenic glutamate receptors (Fig. 6A). Interestingly, only Cluster P3 shows significant enrichment for patients with high Braak stage where 59.44% of the patients in this cluster have been diagnosed with Braak stage 5 or 6 (Fig. 6B–D, Fisher’s exact test *p*-value < 2.2e-09). This enrichment is most significant in BM22 and BM36 brain regions (Fig. 6D,E, and Table 1). Visualization of the expression profiles in Fig. 6A using t-SNE shows that Cluster P1 and Cluster P2 are well separated while Cluster P3 deviates from these clusters (Fig. 6F). This might suggest a transition in the transcriptional state of glutamate receptors in Alzheimer’s patients, which correlates with worse patient outcome. These findings suggest that the down-regulation of glutamate receptors that are chemically inhibited in PhenoCluster 4 can contribute to Alzheimer’s pathogenesis and prognosis through increased angiogenesis.

## Discussion

The multicellular organisation of endothelial cells requires tight coordination of multiple molecular and cellular functions to form a microvascular network. Dysfunctional endothelial cell signalling at the molecular level should manifest in changes in network formation and morphology at the tissue level. Indeed, we illustrate that extensive phenotyping of endothelial networks upon chemical genetic perturbations can provide important insights on mechanisms of action and signalling components affecting the dynamics of self-organising endothelial cells. To our knowledge, our work provides the most comprehensive quantitative analysis of endothelial network phenotypes based on the largest screening dataset employing the tube formation assay.

Quantitative phenotypic profiling of endothelial networks provides significantly more insights than one dimensional scoring of drug effects based on total branching. In particular, we can identify four clusters that increase total branching but have distinct morphologies and are associated with distinct biological mechanisms. For example, the compounds in PhenoCluster 6 resulted in a network with small and thin tubes, which potentially could be due to the cells' inability to form mature tubes. In this cluster, drugs that inhibit ROCK and MLCK have similar network phenotypes. Both ROCK and MLCK have a common downstream mechanism and reduce phosphorylation of myosin light chain. Myosin signalling is involved in many biological processes such as cell shape, motility, and cell–cell adhesion, which are critical for tube formation^35^. Interestingly, statins, which are inhibitors of HMG-CoA reductase, were phenotypically clustered with ROCK and MLCK inhibitors. Statins are FDA-approved drugs for lowering blood cholesterol^36^. However, it is becoming increasingly apparent that Statins have pleiotropic effects independent of its cholesterol-lowering properties. Statins prevent the synthesis of isoprenoid intermediates that are necessary lipid attachments for the post-translational modification of small GTP-binding proteins, including RhoA, the primary activator of ROCK^21^. Therefore, the phenotypic similarity of endothelial network morphology can be used for the classification of drug and genetic perturbation effects on endothelial biology.

CDK2 inhibitors were enriched in PhenoCluster 1, which is characterised by poor network formation (Fig. 2A). This cluster can reflect the reduction of cell motility rather than cell proliferation as it has been shown that tube formation assay is proliferation-independent^8^. In our screen, the cells were fixed after 8 h of drug treatment to capture direct compound effects. Therefore, we assume that the detected effects on the morphology of endothelial network are not due to the change of endothelial cell number during the assay. Supporting our findings, CDK2 has been shown to modulates cell migration^37^.

Multivariate analysis of network phenotypic features revealed that glutamate receptor antagonists are significantly enriched in PhenoCluster 4, which resulted in enhanced network connectivity. This is an interesting finding since glutamate is one of the most prevalent neurotransmitters in the central nervous system, but the link between glutamate receptors and angiogenesis remains unclear. It has previously been shown that endothelial cells express multiple glutamate receptor types, including the Ionotropic glutamate receptors (NMDA and AMPA), and metabotropic receptors. Additionally, they are functional upon the exposure to glutamate^38–40^. Both receptor types were found to have similar endothelial network structure. Ionotropic glutamate receptors are ligand-gated ion channels which open to allow ions such as Na^+^, K^+^, Mg^2+^, and/or Ca^2+^ to pass through the membrane in response to the binding of glutamate^41^. Previous findings provided direct evidence that excessive glutamate leads to increased vascular permeability through activation of NMDA receptors^42^. Thus, we speculate that the angiogenic effect of glutamate receptor inhibition is mediated by the changes in ion transit via the glutamate-activated ion channels.

We verified that the expression of glutamate receptor genes that are known to be antagonised by drugs in PhenoCluster 4 negatively correlates with angiogenic genes and their suppression is associated with worse patient outcome (Fig. 6A,D). The enhanced angiogenesis in PhenoCluster 4 is further supported by the fact that agonists of P2Y, nicotinic acetylcholine and the serotonin HTR1B receptors are also enriched in this cluster. These drugs have been shown to play a proangiogenic role^43-45^. In summary, both tube formation assay and gene expression analysis support an anti-angiogenic role of GRM1, GRIA2, and GRIN3A genes.

The anti-angiogenic effect of some glutamate receptors might initially seem contradictory to the fact that the glutamate receptor antagonist memantine is used to normalise glutamate neurotransmission in Alzheimer’s patients^46^. However, our multi-parametric analysis shows that memantine is in PhenoCluster 5. We found that GRIN1 and GRINA that are antagonised by multiple drugs in PhenoCluster 5 positively correlate with pro-angiogenic genes. Furthermore, GRINA and GRIN1 expression in patient cluster P3 is associated with worse patient outcome, which is the opposite trend to glutamate genes that are targeted by drugs in PhenoCluster 4. However, more experiments are needed to validate the role and the effect of glutamate receptor antagonists in PhenoCluster 5, such as memantine, on endothelial cell function, and how the change in network topology in vitro corresponds to vascular morphology in vivo.

Our integrative analysis of glutamate receptor genes that are identified by our phenotypic screen shows that expression patterns of glutamate receptors in Alzheimer’s patients fall into two groups. One limitation of our meta-analysis is that we cannot determine whether glutamate receptor genes identified through our phenotypic screen are expressed by endothelial cells based on bulk gene expression of brain tissue. Nonetheless, the striking similarity of glutamate receptor profiles based on their network morphology in vitro and their transcriptional profiles in Alzheimer’s patients strongly supports a differential role of glutamate receptors in angiogenesis. Collectively, our results can have important implications when targeting the glutamatergic system in Alzheimer’s patients.

Endothelial and neuronal cells have been shown to share multiple regulatory proteins that guide the motility and growth of both neuronal and endothelial cells during vascular development, ^47^. Furthermore, neurotransmitters, such as glutamate and serotonin, have also been shown to play a role in neuronal guidance during vascular development^48^. In adulthood, many clinical studies reported the role of neurotransmitters such as serotonin and dopamine in tumour angiogenesis^49,50^. Given the strong anatomical and functional relationship between endothelial cells and neural cells such as astrocytes and glial cells in the Blood Brain Barrier, glutamate might play a role in endothelial cell biology where the blood brain barrier is dysfunctional in diseases such as vascular dementia or Alzheimer’s disease^51,52^. Our findings highlight a potential reciprocal relationship between dysregulated neurotransmission and angiogenesis in the context of Alzheimer’s disease. Notably, pro-angiogenic stimuli can have dual actions on the functionality of angiogenesis depending on their abundance and the microenvironment. For example, even though VEGF is considered as a pro-angiogenic factor, excessive levels of VEGF would result in leaky and disorganized vessels with less branching^53,54^. Therefore it is vital to perform in vivo testing to confirm how the identified drug candidates influence angiogenesis in vivo and elucidate the molecular mechanisms underlying this relationship.

In conclusion, we show that accounting for different aspects of microvascular network morphology is important to understand the biological mechanisms underlying the behaviour of complex biological systems. Our approach of multi-parametric phenotyping can be applied to other biological network systems such as neural circuits or mammary gland morphogenesis. As endothelial cells are interwoven in almost all tissues, our work provides a valuable resource for drug discovery studies aimed at targeting angiogenic-related diseases. We envision that our multiparametric phenotyping can significantly improve angiogenic drug development and advance our understanding of endothelial microvascular network biology.

## Methods

### Dataset

The dataset used in this study was generated by an image-based screen of 1,280 compounds based on the LOPAC library as described previously^19^. Briefly, HUVEC endothelial cells were cultured on top of Matrigel after adding compounds and incubated at 37 °C, 5% CO_2_ for 8 h. Then cells were fixed and stained with Phalloidin and imaged by the high content imaging system Operetta from Perkin Elmer.

### Image analysis

Image analysis was performed using ImageJ and MatLab.

*Classification of network elements:* Angiogenesis Analyzer (ImageJ macro) was used to segment network structure and classify its elements^55^. Briefly, the network skeleton is used to extract tubes and branching points (nodes) which are classified into (1) segments: tubes that are connected to the rest of the network from both sides, (2) twigs: tubes that are linked to the rest of the network from one side and (3) isolated tubes: tubes that are not connected to the rest of network, and (4) master segments: segments that are connected to other segments from both sides^55^. Similarly, nodes are also subclassified into (1) junctions: nodes linking two or more tubes, (2) extremities: nodes that are linked to only one tube and (3) master junctions: two or more junctions in close proximity to each other. The algorithm was extended to extract detailed features for each of these elements where various statistics were computed including mean, standard deviation, number and total of each element length or area.

*Network topology characterisation:* Measurements from graph theory were used to quantify vascular network topology. The vascular network was represented as a graph where nodes in the endothelial network correspond to a set of vertices and tubes to a set of edges in the graph. Different centrality metrics of the graph were computed including betweenness, closeness and shortest paths.

Voronoi tessellation was defined based on the branching points. Voronoi diagram partitions a plane with a set of seed points into convex polygons such that each polygon contains exactly one generating point and every point in a given polygon is closer to its seed point than to any other. The average and standard deviation of the resulting polygons sizes can reflect the homogeneity of nodes distribution.

Fractal dimension and polygon size are significantly different from the control-like cluster in all other PhenoClusters (Kolmogorov–Smirnov test *p*-value < 0.0002). However, these measures are highly correlated with well-understood variables. For example, the fractal dimension is highly correlated with total tube length (Supplementary Fig. 3A–C). Voronoi polygon area is highly correlated with mean tube length based on our dataset (Pearson coefficient = 0.58 and Supplementary Fig. 3D–F). Nonetheless, these measures might vary from one dataset to another and therefore might allow detection of unexpected phenotypes.

Texture measures based on Grey Level Co-occurrence Matrix (GLCM) were used to quantify intensity variation in Phalloidin.

### Clustering of phenotypic data

Empty wells and wells with artefact were identified manually and filtered from further analysis. All data were normalised to plate control by subtracting the mean of DMSO-profiles and dividing by the standard deviation of DMSO-profiles for that plate. Then compound replicates were averaged. All the resulting data were scaled between 0 and 1. Then t-SNE was used to visualise the data in two dimensions. Drugs were then clustered using hierarchical clustering based on ward linkage and Euclidean distance.

Notably, assessment of individual features can be difficult as different phenotypic features are driving our clustering. For example, estimation of the probability distribution of individual features, such as total tube length and mean tube length, does not fully separate the different clusters (Supplementary Fig. 3). This demonstrates the importance of multiparametric analysis to obtain a better characterisation of drug effects.

### Drug library annotation

The drug information was downloaded from the DrugBank Database in June 2017. Each drug was annotated with multiple mechanisms of action. Additionally, as drugs varied in the annotated mechanism of action specificity, drugs were annotated with the more general as well as the specific mechanism of action. For example, drugs that were annotated as HTR1A receptors were also annotated with the more general mechanism of action HTR receptors.

### Glutamate receptors target identification

Target genes of glutamate receptor antagonists in PhenoCluster 4 and 5 were identified based on DrugBank database. Additionally, we used the Sigma website which listed many potential targets. To increase the confidence of the association between gene-drug phenotype, only genes that are targeted by at least two drugs in the same PhenoCluster based on Sigma were indicated in Fig. 5B (Supplementary Table 5).

### Analysis of Alzheimer’s expression data

We used the publicly available Mount Sinai Medical Center Brain Bank (MSBB) gene expression dataset of 301 Alzheimer’s patients^56^. The dataset is composed of brain samples with different Braak stages (1–6) that are based on the topographical distribution of neurofibrillary tangles and neuropil threads where stage 6 is the most severe form of the disease^33^.

Data pre-processing: Genes that have a low expression on average were filtered where a cut-off of 1.00 had been estimated based on the distribution of the average expression of all genes. Then the data were scaled between 0 and 1.

Pro-angiogenic gene analysis: To estimate which pro-angiogenic genes are differentially expressed in patients with a higher Braak stage we estimated the fold change of these genes in each brain region. Specifically, we computed the difference between average gene expression values in patients with Braak stage 5 or higher, and the average expression values in patients with Braak stage 3 or lower.

Patient profiles were clustered using hierarchical clustering with ward linkage and Euclidean distance.

## Supplementary information

Supplementary Figure 1.

Supplementary Figure 2.

Supplementary Figure 3.

Supplementary Table 1.

Supplementary Table 2.

Supplementary Table 3.

Supplementary Table 4.

Supplementary Table 5.

Supplementary Table 6.

## Data Availability

The data generated in this study is available in Supplementary Table 6.
