# Supplementary figures and images for: Morphological landscape of endothelial cell networks reveals a functional role of glutamate receptors in angiogenesis
Source: Sci Rep. 2020 Aug 14;10:13829. doi: 10.1038/s41598-020-70440-0 (PMC7428010; doi:10.1038/s41598-020-70440-0)

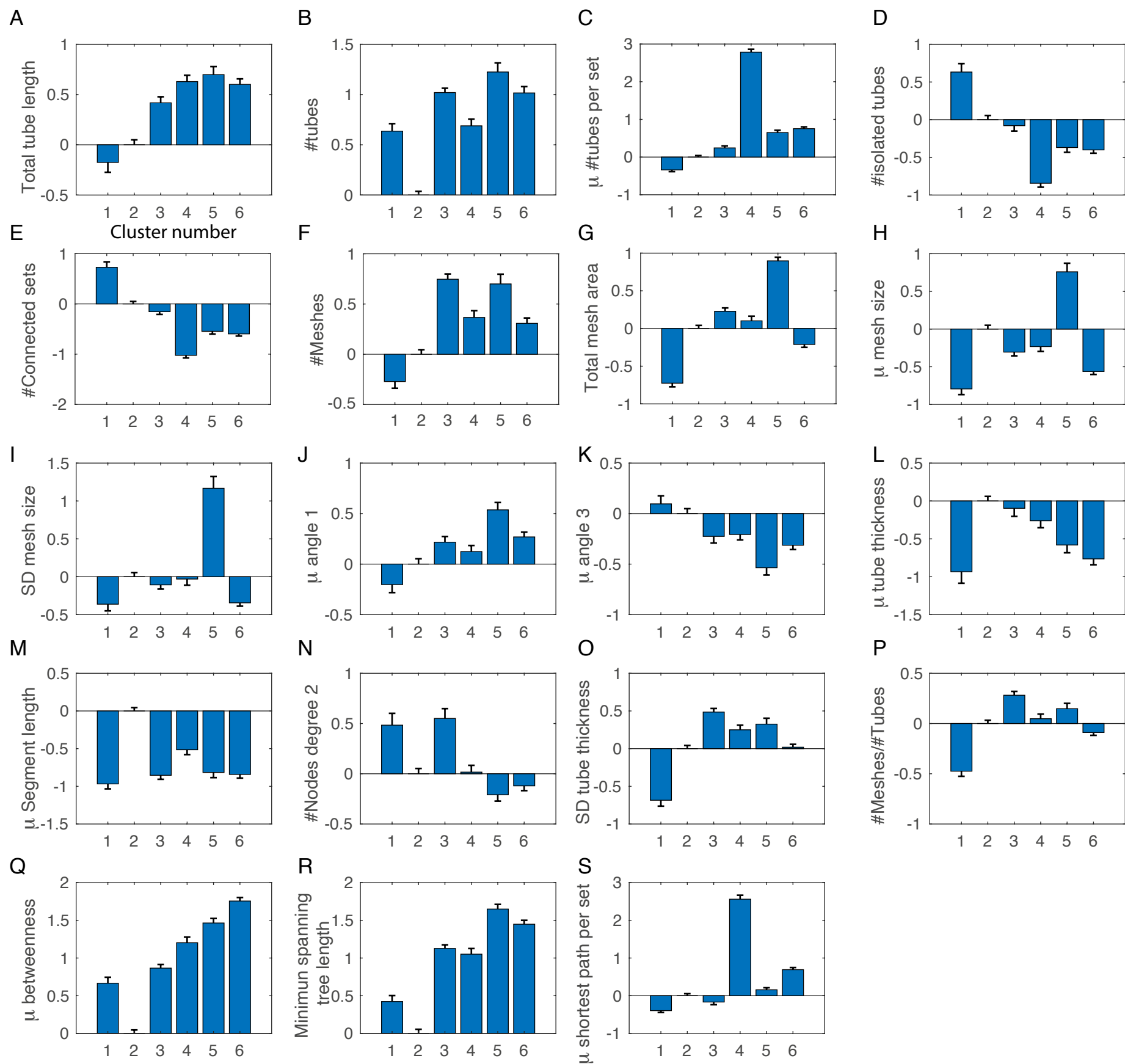

Supplement: Supplementary file 1 — Supplementary Figure 1. [file 41598_2020_70440_MOESM1_ESM.pdf]

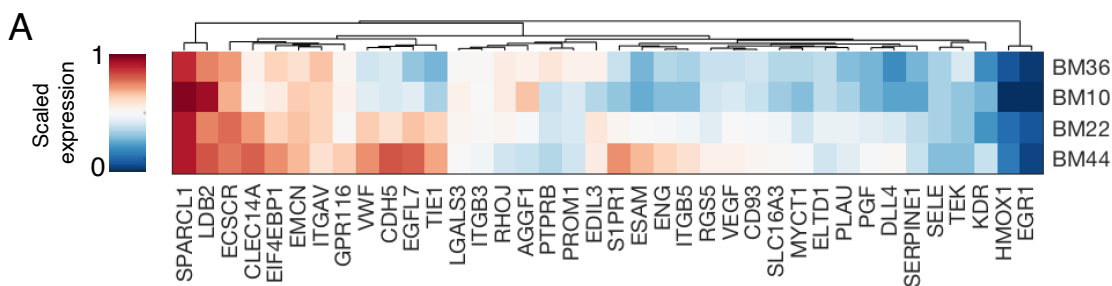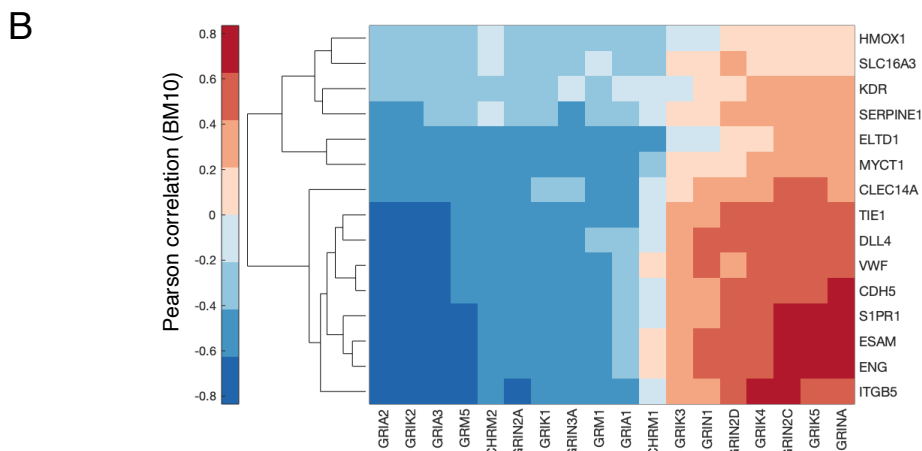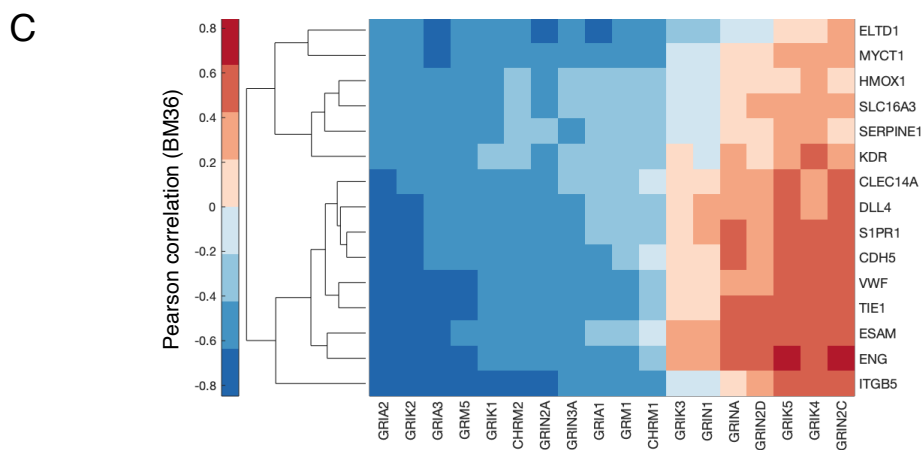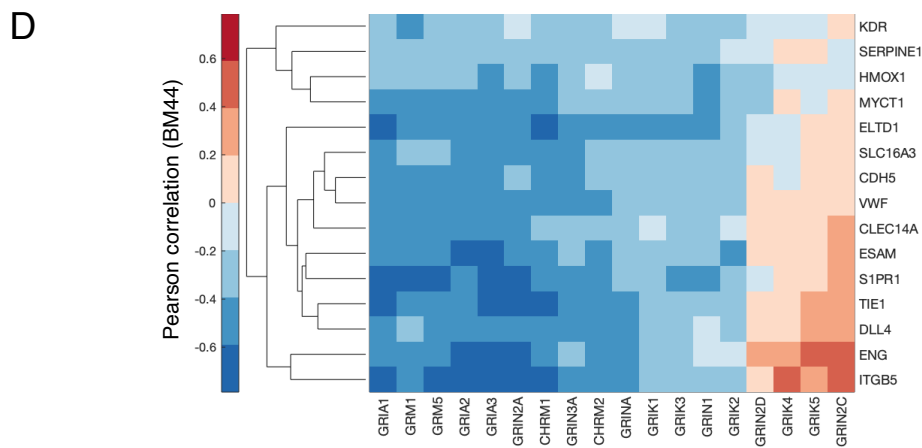

Supplement: Supplementary file 2 — Supplementary Figure 2. [file 41598_2020_70440_MOESM2_ESM.pdf]

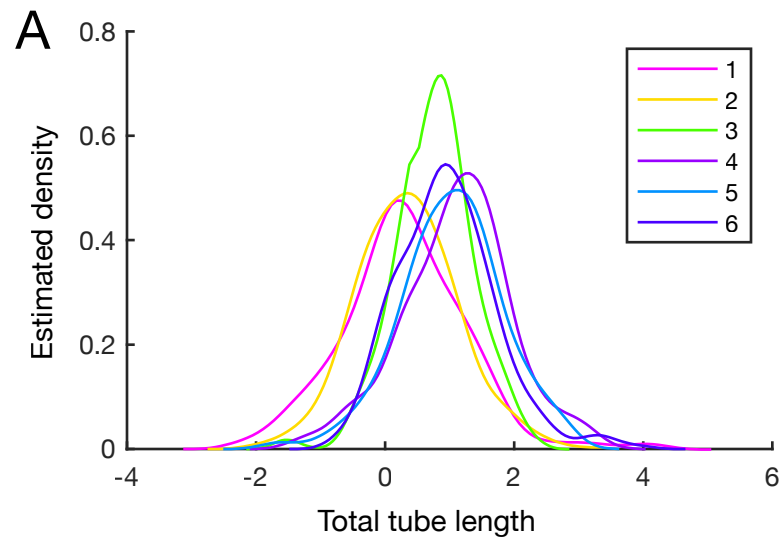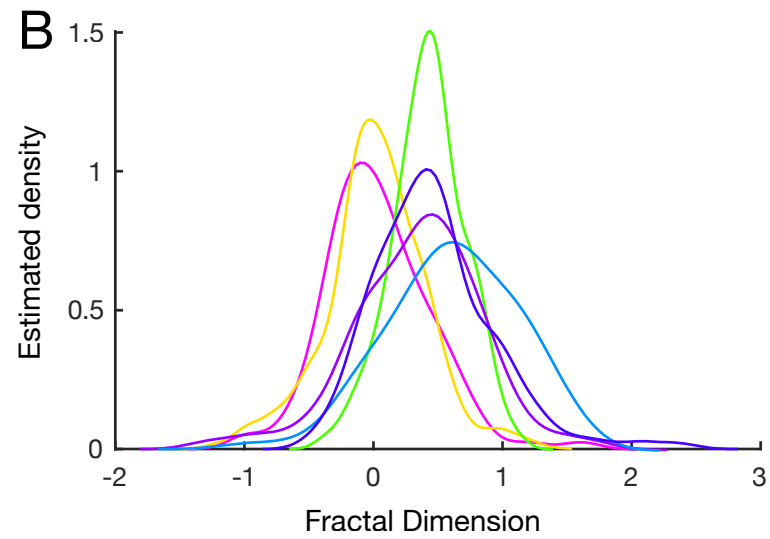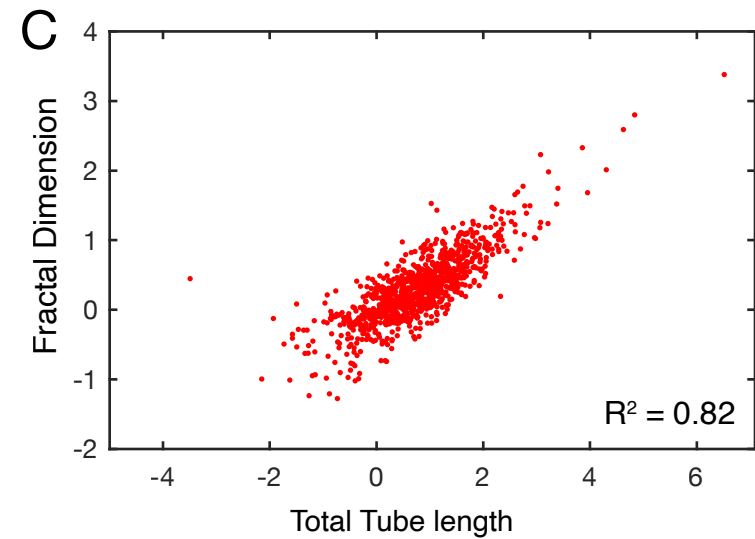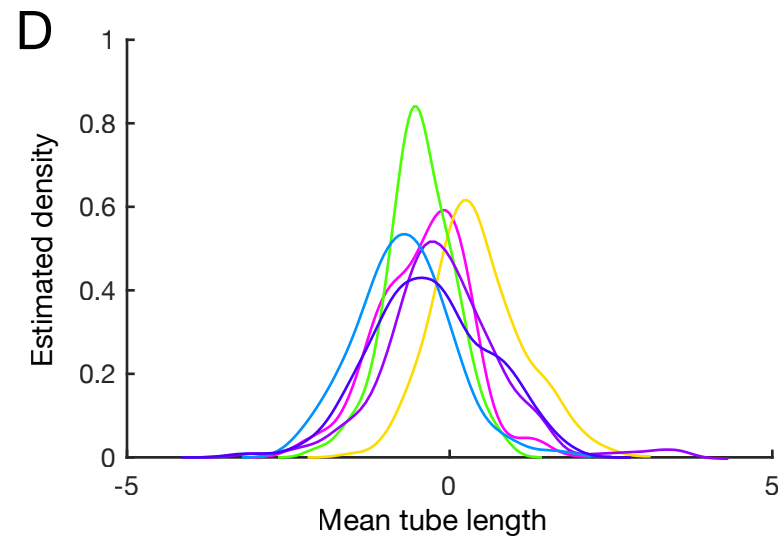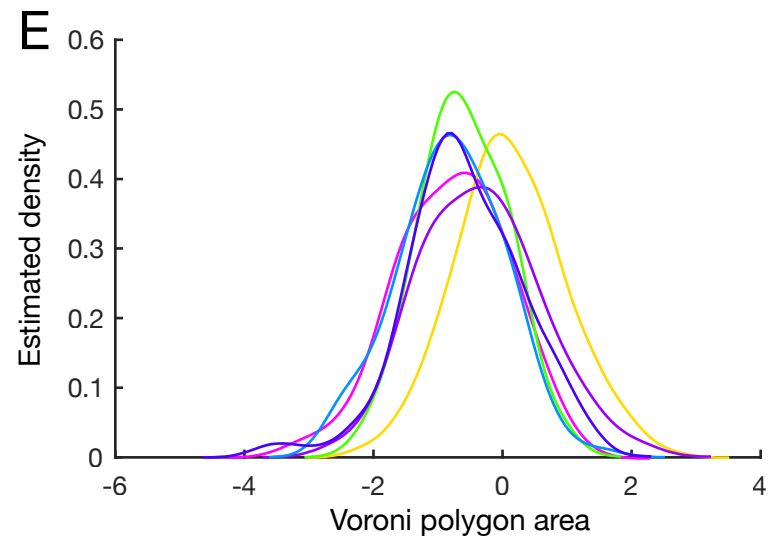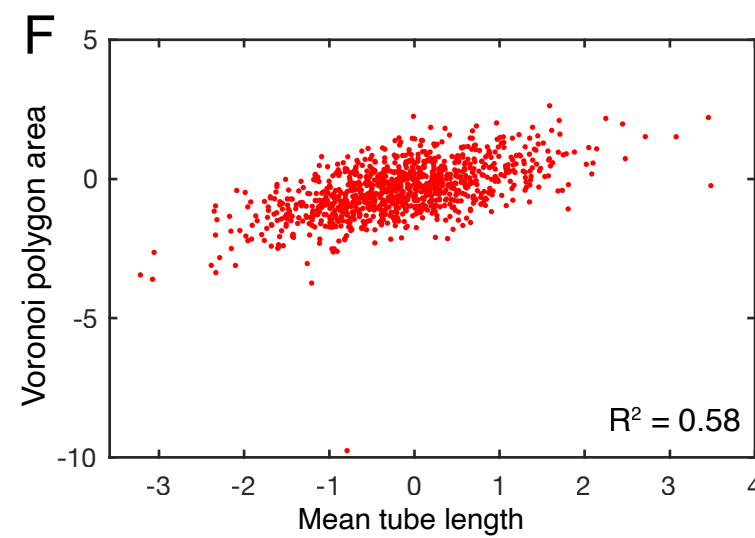

Supplement: Supplementary file 3 — Supplementary Figure 3. [file 41598_2020_70440_MOESM3_ESM.pdf]
